# Evaluation of a digitally enhanced cardiac auscultation learning method: a controlled study

**DOI:** 10.1186/s12909-021-02807-4

**Published:** 2021-07-12

**Authors:** Fred-Cyrille Goethe Doualla, Georges Bediang, Chris Nganou-Gnindjio

**Affiliations:** Faculty of Medicine and Biomedical Sciences, University of Yaoundé I, Yaoundé, Cameroon

**Keywords:** Cardiac auscultation, Electronic stethoscope, Digital-based auscultation, e-learning

## Abstract

**Background:**

Cardiac auscultation remains an efficient and accessible diagnostic tool, especially in resource-limited countries where modern diagnostic devices like cardiac ultrasound are expensive and difficult to access. However, cardiac auscultation skills of medical students and physicians are declining, mainly because of an ineffective teaching method for this technique. The objective of this study is to evaluate the effect of a digitally enhanced cardiac auscultation learning method on participants’ theoretical knowledge and auscultation skills.

**Methods:**

This will be a controlled study with two parallel arms (1:1). Participants (fourth-year medical students) will be divided into two groups: an intervention group (receiving additional lectures, clinical internship and audio listening sessions) and a control group (receiving additional lectures and clinical internship). At the beginning of the study, all participants will undergo a pre-test that consist of two parts: a knowledge assessment based on multiple-choice questions and a skills assessment based on recognition of cardiac sounds from audio files. Thereafter, three specific additional lectures on cardiac auscultation will be delivered and all participants will take part in their official clinical internship. During these clinical internships (eight weeks), participants of the intervention group will be invited to two listening sessions based on five digital recordings of heart sounds. At the end of the clinical internship, all participants will be invited to a post-test to evaluate their knowledge, skills and satisfaction according to their learning method. The main outcome will be the participants’ knowledge progression. The other outcomes will be the participants’ skills progression, participants’ total progression and satisfaction. Data will be collected and analyzed in per protocol.

**Discussion:**

This study could contribute to the development of a learning method that takes into account the advantages of the conventional method and the contribution of digital technology. Positive results could lead to improved cardiac auscultation skills among health professionals, especially in developing countries.

**Trial registration:**

The trial is registered on the Pan-African Clinical Trials Registry (http://www.pactr.org) under unique identification number: PACTR202001504666847, registered the 29 November 2019.

**Supplementary Information:**

The online version contains supplementary material available at 10.1186/s12909-021-02807-4.

## Background

Auscultation is a technique used to diagnose diseases whose pathophysiological mechanisms involve the emission of characteristic sounds by the affected organs [1]. It is based on the use of the stethoscope, invented in 1816 by the French doctor René Laennec [1]. The development of auscultation has been a breakthrough in the diagnosis and management of heart and lung diseases, making it an integral part of clinical examination [2]. Despite the development of many modern diagnostic tools such as cardiac ultrasound, the mastery of auscultation remains a major challenge for health professionals given its effectiveness, efficiency and accessibility, particularly in countries with limited resources [3–6].

However, several studies have shown that the cardiac auscultation skills of medical students and physicians at all levels of training are declining [7–11]. This is explained not only by a decrease in interest in auscultation in favor of new diagnostic tools (echocardiography, MRI, etc.), but above all by an ineffective teaching method for this technique [12, 13].

Indeed, the conventional method of teaching clinical auscultation (lectures associated in a second time with clinical internships) has not evolved for nearly 50 years [12]. Classically, this teaching is done in two phases: (i) a theoretical phase in which the anatomical and physiological basis of the heart as well as the semiological characteristics of cardiac sounds are described and (ii) a practical (clinical) phase in which teachers listen to patients using a conventional stethoscope and identify characteristic sounds that they describe to learners who must then try to recognize the previously identified sounds.

This teaching and learning system may have some limitations, namely: the weak integration between the learning of theoretical knowledge and the acquisition of practical skills (knowledge is acquired long before it is put into practice); the lack of access to patients due to the large number of medical students and the variability of learning due to the diversity of patients and the variable evolution of the symptoms found in these patients [12, 14]. Digitalisation offers new perspectives to auscultation through functionalities like noise reduction, amplification and sound recording. Sound amplification and noise reduction improve sound perception, while recorded sounds can be replayed and shared with others. Consequently, several alternative methods of learning auscultation have been developed to modernize and strengthen this teaching in order to improve the skills of health professionals [12]. A hybrid learning method (conventional method reinforced by the use of digital) could improve the knowledge and skills of health professionals in auscultation and contribute to improve the quality of care, especially in resource-limited countries. The interest of this study is therefore based on the assessment of the relevance of this hybrid learning method and its effects on participants’ knowledge and skills.

## Objectives

### Primary objective

This study aims to evaluate effects of the use of a digitally enhanced auscultation learning method on participants’ cardiac auscultation performance.

### Secondary objectives

 The secondary objectives are to evaluate in both (intervention versus control) groups: (i) the progression of participants’ cardiac auscultation knowledge, (ii) the progression of participants’ cardiac auscultation skills (recognition of sound anomalies), (iii) the total progression of participants’ cardiac auscultation knowledge and skills and (iv) the participant satisfaction.

## Trial design

**Fig. 1:**
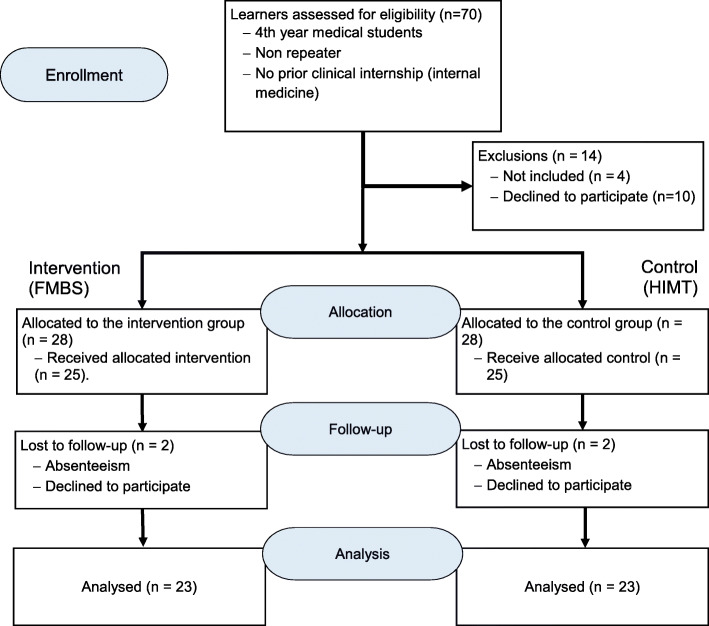
Design of the study

This will be a controlled study with two parallel arms in two centers (Fig. 1). Participants meeting the inclusion criteria will be divided into two groups (1:1): an intervention group (additional lectures associated with a clinical internship and listening sessions based on digital recordings of heart sounds) and a control group (additional lectures with a clinical internship). This study will take place over a six-month period, from the 1st September 2021 to the 1 March 2022 at the Faculty of Medicine and Biomedical Sciences of Yaoundé (FMBS) and at the Higher Institute of Medical Technologies (HIMT). The follow-up of participants from both groups and data collection will take place throughout the study.

## Methods

### Study setting 

This study will take place in two centers: the Faculty of Medicine and Biomedical Sciences of the University of Yaoundé I (FMBS) and the Higher Institute of Medical Technologies (HIMT). These two institutions are medical training schools in Yaoundé, Cameroon.

### Eligibility criteria 

Eligible participants must be medical students newly admitted in to the fourth year of studies and willing to participate in this study. At this stage, they will have completed only nursing training courses.

Participants who did not participate to: (i) additional lectures, (ii) clinical internship, (iii) both listening sessions (based on digital recordings of heart sounds) for intervention group only, (iv) knowledge and skills assessments and (v) those who have withdrawn their consents will be excluded.

Repeaters students will not be included.

### Informed consent 

 Informed consent will be obtained from each participant. This will be done during an interview in which an information sheet and an informed consent form will be explained and given to them.

### Additional consent provisions for collection and use of participant data and biological specimens

Not applicable.

### Interventions

### Explanation for the choice of comparators

The conventional method (lectures and clinical internship) will be chosen as the comparator because it is the most used learning method of cardiac auscultation. This method consists in learning of cardiovascular and respiratory semiology in the third year and in learning of clinical pathology and clinical internships in the fourth year of medical studies respectively.

### Intervention description : intervention group

Three main activities will be organized: additional lectures, clinical internship and listening sessions based on digital recordings of heart sounds.

#### Additional lectures

Three additional lectures will be prepared by research team in agreement with a scientific committee composed of cardiologists. These lectures aim at supporting the normal lectures delivered in these faculties. The first course will focus on the anatomical and physiological bases of the heart, the second on cardiac semiology and the third on cardiac auscultation. These courses will take place at the FMBS and will be delivered in two sessions: the first course (first session) will take place on the same day as the recruitment of participants, the second and third course (second session) will take place two or three days after the first. Each course will be administered using PowerPoint presentation software and will have a duration of one hour (30 min of teaching and 30 min of question-and-answer session). These courses are complementary to other theoretical lectures in clinical semiology which are delivered in the amphitheater or in the clinical departments in which they have an internship and which correspond to the training program of 4th year of medical studies.

#### Clinical internship

Participants in the intervention group will be required to participate in their official clinical internship (internal medicine) as planned by their training school. This internship will take place in all the hospitals in Yaoundé that are accredited by the FMBS, namely the Central Hospital, the General Hospital, the University Hospital Centre and the Jamot Hospital. It will start after the delivery of additional lectures, over a period of two months. The objective is to introduce participants in a practical way to the patient’s cardiac examination (inspection, palpation, percussion and auscultation) and to reinforce their knowledge of cardiac semiology. These objectives will be achieved through activities such as medical observations, daily follow-up of hospitalized patients, practical cardiac semiology sessions and participation in scientific symposia. These activities are most often done under the supervision of a teacher or of an academic elder, a resident or an intern.

#### Listening sessions based on digital recordings of heart sounds

##### Preparation of the material

Five (5) digital audio recordings from real patients will be selected from a cardiac auscultation database designed in Cameroon in 2017 [15]. These recordings were made using a Littmann© 3200 electronic stethoscope and the resulting audio files were annotated using Audacity © software under the supervision of a cardiologist and then stored on a computer in WAV format.

Each recording corresponds to a specific cardiac sound and lasts for one (1) minute; the target cardiac sounds are as follows: heart sounds B1 and B2, aortic stenosis murmur, third heart sound (B3), fine crackles of heart failure and atrial fibrillation murmur. The selected recordings will be stored on a computer.

##### Listening sessions

Two listening sessions of these digital recordings will be held in an FMBS classroom. Each session will be held for one hour. Each session will be organized in 3 parts: 10 min for the introduction of the activity and the presentation of tools; 40 min for listening during which each participant will have access to the 5 selected digital audio recordings of heart sounds and 10 min for feedback (collection of participants’ opinions and impressions). These recordings will be played back in a loop (series of 5) to the participants using a computer connected to loudspeakers and the Audacity© software. Four series of 10 min will be made. During these series, each recording will be broadcast for 2 min, for a total of 10 min per serie. The first series will be commented by the supervisor to help participants to identify auscultatory anomalies.

A multimedia package containing the additional lectures and the 5 digital audio recordings will be designed and made available to each participant in the intervention group at the end of the second listening session. Participants will be recommended to use this support to independently conduct similar listening sessions as often as they wish. SMS reminders will be sent to participants to encourage them to use this support.

### Intervention description : control group

#### Additional lectures

The training of cardiac auscultation in the control group will be done by the conventional method (association of lectures and a clinical internship). As in the intervention group, participants will also attend three additional lectures on anatomical and physiological bases of the heart, cardiac semiology and cardiac auscultation respectively. These courses will take place at the HIMT and will be delivered in two sessions: the first course (first session) will take place on the same day as the recruitment of participants, the second and third course (second session) will take place two or three days after the first. Each course will be administered using PowerPoint presentation software and will have a duration of one hour (30 min of teaching and 30 min of question-and-answer session). These are courses given in addition to the normal theoretical training program corresponding to the normal 4th year medical studies program. These courses are delivered in addition to the normal theoretical training program corresponding to 4th year medical studies.

#### Clinical internship

Participants in the control group will be required to participate in their official clinical internship (internal medicine) also as planned by their training school. This internship will take place in all the hospitals in Yaoundé that are accredited by the HIMT and will start after the delivery of additional lectures, over a period of two months.

### Evaluation of knowledge and skills

Two evaluations (pre-test and post-test) will be organized during this study in the two groups. Each assessment will consist of two parts: a knowledge (theoretical) assessment based on multiple-choice questions and a skills (practical) assessment based on a test to recognize cardiac sounds from the audio files.

The first evaluation (pre-test, see additional file 1) will take place at the beginning of the study, directly after the recruitment of participants. The knowledge (theoretical) assessment will aim to evaluate participants’ basic knowledge and will be made on the basis of a form consisting of 15 multiple-choice questions (1 point per correct answer) validated by a scientific committee. These questions will focus on the basic anatomy and physiology of the heart, as well as the technique and semiological aspects of cardiac auscultation. The skills (practical) assessment will aim to assess the participants’ ability to recognize characteristics of cardiac sounds from audio recordings. Information on the locations of cardiac sound recording sites will be provided to participants. Participants will be invited to listen to these cardiac sound recordings using a computer connected to a loudspeaker. The five digital audio recordings selected (one normal and four pathological) will be numbered from 1 to 5. The reading sequence will be random (using a randomization tool). Each recording will be played and listened for one minute and two additional minutes will be allowed for reflection on each recording and for writing the response. Dimensions assessed will be the normal or pathological nature of a cardiac sound, timing and description of the abnormality if it is present. Each correct answer will give one point, for a total of five points.

The second evaluation (post-test, see additional file 2) will take place at the end of the participants’ clinical internships, about 60 days after the first. The purpose of this evaluation will be to measure the participants’ progress in terms of knowledge and skills according to their respective learning methods. It will also consist of a series of 20 questions similar to those in the pre-test (15 multiple-choice questions and 5 cardiac sound recognition questions). However, the questions will have a different order in the post-test form and will be defined by drawing of lots. Each question will receive an identifier based on a numeric code ranging from 1 to 15 for multiple-choice questions and from 16 to 20 for cardiac sound recognition questions. Every code will be written on paper and placed in two different ballot boxes. A person independent of the research team will be invited to randomly draw fifteen questions from the first box and fives questions from the second one.

Participant satisfaction (see additional file 3 for the control group and additional file 4 for the intervention group) will be assessed using a form containing questions whose answers will be presented in the form of a four-model Likert scale. The dimensions evaluated will be as follows: the training tools used, the pedagogy implemented and the benefits obtained by participants according to their learning methods.

### Criteria for discontinuing or modifying allocated interventions

This study compares a classic approach to training auscultation students versus a hybrid method which combines listening sessions based on recorded digital heart sounds. It does not involve the administration of drugs that may cause harms or worsening disease. There is therefore no reason to justify the discontinuity or the modification of the intervention for a participant.

### Strategies to improve adherence to interventions

 Strategies to improve adherence to intervention protocols will be based on the use of a tracking sheet containing the phone numbers of each participant, on phone calls made by investigators regularly to invite participants to sessions and to follow up them and on visits (supervision) of their clinical internships. Listening sessions and evaluation will be planned according to the participants’ academic schedules.

### Relevant concomitant care permitted or prohibited during the trial

Participants will be allowed to access all teaching materials in accordance with their learning processes as defined by their respective training schools.

### Provisions for post-trial care

 The study is based on a non-invasive intervention for the participants (lectures, clinical internships, listening sessions). The risks incurred during the process (namely during the clinical internships) will be covered respectively by the participants’ training schools and the accredited hospitals.

### Outcomes

Baseline data will consist of presenting the socio-demographic data of the learners, the pre-test knowledge score, the pre-test skills score, the pre-test total score, the post-test knowledge score, the post-test skills score and the post-test total score.

The primary outcome will be the progression of participants’ knowledge in cardiac auscultation. It will be evaluated on the basis of the difference between the participants’ knowledge score in the post-test and the participants’ knowledge score in the pre-test.

The secondary outcome will be: (i) the progression of participants’ skills in cardiac auscultation (based on the difference between the participants’ skill score in the post-test and the participants’ skill score in the pre-test), (ii) the total progression of participants’ knowledge and skills in cardiac auscultation (based on the difference between the total score obtained by the participants in the post-test and the total score obtained in the pre-test) and (iii) the participants’ satisfaction (Table 1).

**Table 1:** Outcomes

Outcomes	Measurement	Type	Statistical analysis
**Primary outcome**			
Progression of participants’ knowledge in cardiac auscultation.	Difference between knowledge post and pre-test score	Continue	t-test or non-parametric equivalent
**Secondary outcomes**			
Progression of participants’ skills^a^ in cardiac auscultation	Difference between skills^a^ post and pre-test score	Continue	t-test or non-parametric equivalent
Total progression of participants’ knowledge and skills^a^ in cardiac auscultation	Difference between total progression post and pre-test score	Continue	t-test or non-parametric equivalent
Satisfaction	Satisfaction score	Ordinal	Non parametric test

^a^Recognition of cardiac sounds

### Participant timeline

For all groups, enrolment, allocation of participants and pre-test will take place simultaneously at the beginning of the study. Thereafter, the additional lectures will be given one week before the beginning of the clinical internship. The clinical internship will be held over a period of eight weeks.

**Fig. 2:**
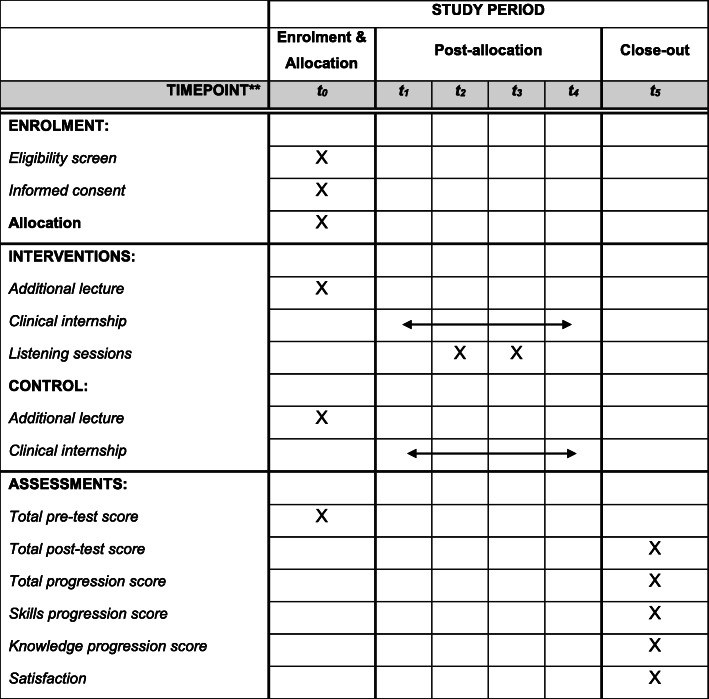
Participant timeline. **t_0_: beginning of the study; t_1_: first week of clinical internship; t_2_: second week of clinical internship; t_3_: fourth week of clinical internship; t_4_: end of clinical internship (eighth week); t_5_: end of the study (close-out)

For intervention group, the first listening session will take place one week after the beginning of the clinical internship, while the second listening is held two weeks after the first session. The post-test will take place at the end of the clinical internship (Fig. 2).

### Sample size

The minimum sample size will be calculated on the basis of a comparison of the average knowledge progression between the two groups. The hypothesis is that participants in the intervention and control groups will have an average of 30 % correct answers in the pre-tests and a 50 % increase in the intervention group compared to 10 % in the control group in the post-tests, i.e., an expected difference of 40 % between the two groups. For a power of 80 %, a ratio of 1:1 per group and an alpha error of 5 %, the minimum sample size will be 46 participants (23 per arm) [16]. Given the high risk of a refusal to participate, absenteeism and loss of sight, the minimum sample size will be increased by 20 %, for a total of 56 participants.

### Recruitment

The potentially eligible participants at the intervention center are estimated at 100, while those at the control center are estimated at 50. Those participants will be approached by an investigator in their training schools. The potential benefits of participating in this study will be explained to ascertain the participants’ interest.

### Assignment of interventions: allocation

#### Sequence generation

We have a potential risk of communication between participants in the intervention and control groups if they are in the same institution. To avoid this, participants in the control group will be selected only at HIMT while participants in the intervention group will be selected at FMBS. These two institutions are geographically distant and they organize their clinical internships in several hospitals at the same time.

#### Concealment mechanism

Not applicable.

#### Implementation 

All participants will be invited to take part in three additional lectures and a pre-test prior to their official clinical internships. SMS and phone calls will be used during clinical internships to follow up all participants. Participants in the intervention group will receive additional SMS reminders to encourage them to participate in the supervised listening sessions and to conduct their own listening sessions. Participants in the intervention and those in the control group will perform a post-test at the end of their clinical internship.

## Assignment of interventions: blinding

### Who will be blinded

Participants in intervention group and in control group will be selected from two different medical schools respectively. These medical schools organize also their clinical internships in several hospitals. Therefore, participants will remain unaware of whether they are included in the intervention or control group. Teachers and other clinical internship supervisors will not be informed of the group to which each participant belongs because the intervention will be carried out by the research team directly. The evaluation of the intervention and the analysis of the data will be done by the research team.

### Procedure for unblinding if needed

Not applicable.

## Data collection and management

### Plans for assessment and collection of outcomes 

Data will be collected using forms containing 20 questions each. These forms (self-administered) will be given to all participants who agree to participate in this study. The first fifteen (15) multiple-choice questions will assess theoretical knowledge and the last five (5) questions will assess practical skills based on the listening of cardiac sound recordings. Completing this questionnaire will enable to obtain for each participant, a total score, a knowledge score and a skills score during the pre-test and the post-test.

## Plans to promote participant retention and complete follow-up 

Enrolled participants of each center will be followed during the entire study period. The interest for the study will be maintained through phone calls, messages and visits during their clinical internship.

### Data management 

The data will be entered electronically using Epi Data v3.1 software. Data integrity will be enforced through a double data entry. All forms and materials related to the study will be stored in a computer with restricted access. A complete backup will be performed every month.

### Confidentiality

No personal information about enrolled participants will be shared before, during, and after the trial.

### Plans for collection, laboratory evaluation and storage of biological specimens for genetic or molecular analysis in this trial/future use

Not applicable.

### Statistical methods

#### Statistical methods for primary and secondary outcomes

The use of statistical methods will be based essentially on the comparison of participants from the two groups according to defined outcomes. The average knowledge progression of participants in the intervention group will be compared against those in the control group using Student’s t-test if the variables follow the normal distribution or by a non-parametric test if not. The average skill progression and the average total progression in knowledge and skills (based on the total progression score) of the intervention group will be compared against those of the control group using a Student’s t-test if the variables follow the normal distribution or by a non-parametric test if not. The other continuous variables (the pre-test knowledge score, the pre-test skills score, the pre-test total score, the post-test knowledge score, the post-test skills score and the post-test total score.) will also be compared using parametric or non-parametric tests. The satisfaction of all participants (ordinal variable) will be analysed using a non-parametric test.

### Interim analyses

Analyses will only be done at the end of the study.

### Methods for additional analyses (e.g. subgroup analyses)

No further analysis will be done.

### Methods in analysis to handle protocol non-adherence and any statistical methods to handle missing data

Since the primary outcome measures the total progression of participants, it is necessary for each participant to go through the entire process. Participants with missing data will be excluded from the study.

### Plans to give access to the full protocol, participant level-data and statistical code

The access to the full protocol will be granted through a publication in a scientific journal.

### Oversight and monitoring

#### Composition of the coordinating center and trial steering committee

This study will be coordinated by the research team. This team is composed of 5 people and will be responsible for the main activities of the project: writing the protocol, planning and monitoring of the study, allocation and recruitment of participants, implementation of interventions, data collection, analysis of results, writing article and monitoring of publication in a scientific journal.

#### Composition of the data monitoring committee, its role and reporting structure

Two members of the research team will be permanently responsible for data collection. However, monthly meetings are planned to review the data collected.

### Adverse event reporting and harms

Not applicable.

### Frequency and plans for auditing trial conduct

All processes related to the conduct of the study will be audited by the research team on a daily basis. Monthly meetings are planned to review the progress of the study. In any case, in accordance with ethical clarity, one or two audits are planned by the regional ethics and research committee.

### Plans for communicating important protocol amendments to relevant parties (e.g. trial participants, ethical committees) 

In case of changes in the protocol, the research team is committed to submit a revised version of the ethics committee and to the Pan-African Trial Registry.

### Dissemination plans 

Four methods of dissemination will be used: (i) feedback sessions to participants, (ii) reports being submitted to faculties/universities and to Ministry of Higher Education, (iii) presentations at national and international scientific congresses and (iv) the publication in an international journal.

## Discussion

The performance of health professionals and medical students in clinical auscultation has declined over the years [7–10, 12, 13]. This decline is due to several factors, including the emergence of new diagnostic tools such as cardiac ultrasound and the ineffective teaching of clinical auscultation [7–10, 12, 13, 17].

Several learning methods have been developed to improve clinical auscultation skills of medical students and health professionals. The most common are: (i) the use of the patient simulator, (ii) the use of specialized software, (iii) the use of mobile applications and (iv) the use of devices such as the electronic stethoscope or ultrasound stethoscopes [18–29].

Patient simulators aims to facilitate learning of auscultation while overcoming some of the limitations of bedside teaching (variability of symptoms, high student-patient ratios, etc.) [18, 30]. This learning method is based on the use of a life-size manikin capable of generating synthetic cardiac sounds [12, 18, 20]. Patient simulators can improve the knowledge and skills of the learners (up to 30 %) by allowing them to listen repeatedly to cardiac sounds in conditions similar to those of the clinical environment [12, 18, 20, 31]. However, it does not allow an autonomous learning of auscultation (the manikin is often kept in the faculty). Specialized software aims to improve auscultation skills by reflecting the examination process of patients and developing learners’ integration of eyes and ears findings [19, 24, 25, 32]. This method consists of real patient’s examination filmed at the bedside, coupled with recorded cardiopulmonary sound [12, 32, 33]. Depending on the software used, a progression up to 70 % of the learners’ knowledge and skills can be observed [24, 25]. Mobile application and computer-based independent learning methods aim to facilitate the acquisition of auscultation knowledge and skills, while preserving the learner’s autonomy [14, 28, 34, 35]. It is based on the use of mobile applications or multimedia supports (CDs) which allow access to cardiopulmonary sounds and lectures [36]. The effects of this method on the knowledge of the learners are similar to those of lectures [8, 12]. However, the use of this method can reduce the teaching burden in medical schools when compared with conventional lectures [12, 29, 35]. The electronic stethoscope is often associated with software to visualize the phonograph [37]. It offers the possibility to record cardiopulmonary sounds of real patients that can be shared with one or more students or used as a teaching tool for other learning methods based on listening to audio files [37]. Moreover, the use of an electronic stethoscope instead of a traditional stethoscope during clinical internships can improve learners’ skills (up to 51.2 %) [38–40].

Digitally enhanced cardiac auscultation is designed to reinforce the classical learning method (theoretical course plus clinical internship) by adding specific lectures and supervised listening sessions based on digital recordings of heart sounds. Specific lectures will provide the background needed by learners to integrate the skills that will be acquired during clinical internships. The supervised listening sessions will provide a better understanding of the specific characteristics of cardiac sounds on the one hand and will guarantee a minimum number of repetitive listening sessions to improve the recognition of cardiac sounds on the other hand. Additional unsupervised listening sessions are recommended to complement the supervised sessions. In our approach, the absence of video content as available in some specialized software is balanced by clinical internships during which the learner will perform the cardiac examination in a practical way.

This study could contribute to the development of a learning method that takes into account the advantages of the conventional method and the contribution of digital technology. Its first added value could be learning flexibility. The learner could conduct listening sessions independently and improve his skills without depending on a patient or a teacher. The second added value could be the reinforcement of knowledge, through all the lectures and an improved integration between knowledge and skills in auscultation. Finally, the acquisition of skills related to the use of digital (electronic stethoscope or associated software) in the context of recording and sharing of cardiac sounds.

## Trial status

The recruitment of participants will begin on 1st September 2021 (start of the 2021–2022 academic year for FMBS and HIMT). Trial unique identification number: PACTR202001504666847.

## Supplementary Information

**Additional file 1.** Pretest questionnaire; questionnaire to assess participants' knowledge and skills at the beginning of the study. File format: docx.

**Additional file 2.** Posttest questionnaire; questionnaire to assess participants' knowledge and skills at end of the study. File format: docx.

**Additional file 3.** Satisfaction questionnaire (control group); questionnaire to assess participants' satisfaction in the control group regarding their learning methods. File format: docx.

**Additional file 4.** Satisfaction questionnaire (intervention group); questionnaire to assess participants' satisfaction in the intervention group regarding their learning methods. File format: docx.

## Data Availability

All data generated and analyzed during this study will be available from the corresponding author upon reasonable request.

## Abbreviations

FMBS: Faculty of Medicine and Biomedical Sciences
HIMT: Higher Institute of Medical Technologies
