# Supplementary material for: Evaluation of a digitally enhanced cardiac auscultation learning method: a controlled study
Source: BMC Med Educ. 2021 Jul 12;21:380. doi: 10.1186/s12909-021-02807-4 (PMC8273941; doi:10.1186/s12909-021-02807-4)
Supplement: Supplementary file 2 — Additional file 2. Posttest questionnaire; questionnaire to assess participants' knowledge and skills at end of the study. File format: docx. [file 12909_2021_2807_MOESM2_ESM.docx]

Evaluation of a digitally enhanced cardiac auscultation learning method: a controlled study

Evaluation questionnaire: post test

**Section 1: Identification of participants**

**Id : _ _ _ _**

**Have you completed your clinical internship in internal medicine during this academic year (2020-2021) ?**

Yes No

**Section 2: Theoretical knowledge**

| **Q1. Regarding cardiac auscultation :**  *Tick one or more correct answers*   - It must be dynamic - It must be systematised - It does not require any special preparation - All answers are correct | **Q2. For the 3^rd^ heart sound (B3) :**  *Tick one or more correct answers*   - It is diastolic - It is always pathological - It is often associated with heart failure - All answers are correct |
| --- | --- |
| **Q3. The following valves are known as atrioventricular valves except :**  *Tick one or more answers*   - The tricuspid valve - The mitral valve - The aortic valve - The pulmonary valve | **Q4. The cardiac cycle can be described in the following order:**  *Tick the correct answer*   - Contraction, ejection, relaxation, filling - Filling, ejection, contraction, relaxation - Ejection, relaxation, filling, contraction - Relaxation, ejection, filling, contraction |
| **Q5. The first heart sound (B1) marks :**  *Tick the correct answer*   - Atrioventricular valve closure - Closure of the sigmoid valves - The passage of blood through the chambers of the heart - No response | **Q6 What do you think is the pathway of the heart's nerve impulses?**  *Tick the correct answer*   - Sinus node, atrioventricular node, His bundle, Purkinje network - Sinus node, His bundle, Purkinje network, atrioventricular node - Atrioventricular node, sinus node, His bundle, Purkinje network - No correct answer |
|  | |
| **Q7. In your opinion, how many conventional cardiac auscultation sites are there?**  *Tick the correct answer*   - 2 - 6 - 7 - 4 | **Q8. In your opinion, the sub-crepitus rales of heart failure :**  *Tick one or more correct answers*   - Reflects flooding of the pulmonary alveoli - The following are described as "rising tide - Get along better at the end of the breath - All proposals are correct |
| **Q9. Which of the following can be used to determine the time of a breath :**  *Tick one answer only*   - Heart rate - Radial pulse - Respiratory rate - Blood pressure | **Q10. In your opinion, the heart is located in :**  *Tick one or more correct answers*   - The posterior mediastinum - The anterior mediastinum - The middle mediastinum - No proposal |
| **Q11. Blows are characterised by the following items except :**  *Tick one answer only*   - The seat - Duration - The intensity - The stamp | **Q12. the parts of the conventional stethoscope are the following except :**  *Tick only one correct answer*   - Ear tips - The lyre - The command centre - No proposal |
| **Q13. the envelopes of the heart are as follows (from inside to outside):**  *Tick one or more correct answers*   - Endocardium - myocardium - pericardium - Myocardium - endocardium - pericardium - Endocardium - pericardium - myocardium - Pericardium - endocardium - myocardium | **Q14 The bell of a conventional stethoscope :**  *Tick one or more correct answers*   - Allows the perception of low frequency sounds - Allows the perception of intermediate frequency sounds - Allows the perception of high frequency sounds - No correct answer |

**Q15. Regarding innocent breaths :**

*Tick one or more correct answers*

- They are always diastolic
- They may be associated with a physiological condition
- They are diffuse
- They do not radiate
- All answers are correct

**Section 3: Recognition of cardiopulmonary sounds**

*You will be asked to listen to five successive sounds from the auscultation of real patients. For each sound, you will have 1 minute to listen and 2 minutes to think about the questions below.*

| **Q16.1. Sound No. 1 is :**  *Tick one answer only*   - Normal - Pathological   **Q16.2. If pathological, at what time is this anomaly perceived?**  *Tick one answer only*   - Systole - Diastole - Inspiration - Expiry   **Q16.3. In your opinion, which auscultatory abnormality is it?**  *_______________________________________* | **Q17.1. Sound No. 2 is :**  *Tick one answer only*   - Normal - Pathological   **Q17.2. If pathological, at what time is this anomaly perceived?**  *Tick one answer only*   - Systole - Diastole - Inspiration - Expiry   **Q17.3. In your opinion, which auscultatory abnormality is it?**  *_______________________________________* |
| --- | --- |
| **Q18.1. Sound No. 3 is :**  *Tick one answer only*   - Normal - Pathological   **Q18.2. If pathological, at what time is this anomaly perceived?**  *Tick one answer only*   - Systole - Diastole - Inspiration - Expiry   **Q18.3. In your opinion, which auscultatory abnormality is it?**  *_______________________________________* | **Q19.1. Sound No. 4 is :**  *Tick one answer only*   - Normal - Pathological   **Q19.2. If pathological, at what time is this anomaly perceived?**  *Tick one answer only*   - Systole - Diastole - Inspiration - Expiry   **Q19.3. In your opinion, which auscultatory abnormality is it?**  *_______________________________________* |
| **Q20.1. Sound No. 5 is :**  *Tick one answer only*   - Normal - Pathological   **Q20.2. If pathological, at what time is this anomaly perceived?**  *Tick one answer only*   - Systole - Diastole - Inspiration - Expiry   **Q20.3. In your opinion, what is the auscultatory abnormality?** *_____________________* |  |
