# Supplementary material for: Evaluation of a digitally enhanced cardiac auscultation learning method: a controlled study
Source: BMC Med Educ. 2021 Jul 12;21:380. doi: 10.1186/s12909-021-02807-4 (PMC8273941; doi:10.1186/s12909-021-02807-4)
Supplement: Supplementary file 3 — Additional file 3. Satisfaction questionnaire (control group); questionnaire to assess participants' satisfaction in the control group regarding their learning methods. File format: docx. [file 12909_2021_2807_MOESM3_ESM.docx]

Evaluation of a digitally enhanced cardiac auscultation learning method: a controlled study

Evaluation questionnaire: satisfaction (control)

**Section 1: Identification of participants**

**Id : _ _ _ _**

*For each item below, please select the answer that most closely matches your learning experience in auscultation.*

**S2. Learning method**

| S1Q1. The teaching materials provided during my learning were appropriate. | | | |
| --- | --- | --- | --- |
| - Strongly disagree | - Somewhat disagree | - Somewhat agree | - Strongly agree |

| S1Q2. I am satisfied with the pedagogical tools used in my learning. | | | |
| --- | --- | --- | --- |
| - Strongly disagree | - Somewhat disagree | - Somewhat agree | - Strongly agree |

**S3. Pedagogy**

| S2Q1. The learning method used was easy to put into practice. | | | |
| --- | --- | --- | --- |
| - Strongly disagree | - Somewhat disagree | - Somewhat agree | - Strongly agree |

| S2Q2. The objectives to be achieved were clearly formulated at the beginning of my learning. | | | |
| --- | --- | --- | --- |
| - Strongly disagree | - Somewhat disagree | - Somewhat agree | - Strongly agree |

| S2Q3. Theoretical courses have been sufficiently developed. | | | |
| --- | --- | --- | --- |
| - Strongly disagree | - Somewhat disagree | - Somewhat agree | - Strongly agree |

| S2Q4. The clinical internship has been sufficiently developed. | | | |
| --- | --- | --- | --- |
| - Strongly disagree | - Somewhat disagree | - Somewhat agree | - Strongly agree |

| S2Q5. The theoretical courses and the clinical internship were harmoniously adjusted. | | | |
| --- | --- | --- | --- |
| - Strongly disagree | - Somewhat disagree | - Somewhat agree | - Strongly agree |

**S3. Benefits**

| S3Q1. My learning method met my expectations. | | | |
| --- | --- | --- | --- |
| - Strongly disagree | - Somewhat disagree | - Somewhat agree | - Strongly agree |

| S3Q2. I can recommend this learning method to my colleagues. | | | |
| --- | --- | --- | --- |
| - Strongly disagree | - Somewhat disagree | - Somewhat agree | - Strongly agree |

| S3Q3. I have achieved the goals that I had set for myself at the beginning of my learning. | | | |
| --- | --- | --- | --- |
| - Strongly disagree | - Somewhat disagree | - Somewhat agree | - Strongly agree |

| S3Q4. I am satisfied with my learning of auscultation. | | | |
| --- | --- | --- | --- |
| - Strongly disagree | - Somewhat disagree | - Somewhat agree | - Strongly agree |

| S3Q5. I feel the need for additional training in auscultation. | | | |
| --- | --- | --- | --- |
| - Strongly disagree | - Somewhat disagree | - Somewhat agree | - Strongly agree |
